# The role of the urologist in managing high flow priapism

**DOI:** 10.1038/s41443-025-01017-6

**Published:** 2025-02-05

**Authors:** Murat Dursun, Arif Kalkanlı, Seyfettin Anıl Tantekin, Ahmet Halil Sevinç, Turgay Kaçan, Celal Caner Ercan, Ateş Kadıoğlu

**Affiliations:** 1 Istanbul University, Faculty of Medicine, Department of Urology, Section of Andrology, Istanbul, Turkey; 2 Taksim Training and Research Hospital, Department of Urology, Istanbul, Turkey; 3 Istanbul University, Faculty of Medicine, Department of Urology, Istanbul, Turkey; 4 Istanbul University, Faculty of Medicine, Department of Radiology, Istanbul, Turkey

**Keywords:** Sexual dysfunction, Diagnosis

## Abstract

High-flow priapism (arterial) is a prolonged erection caused by irregular cavernous arterial flow, often resulting from blunt perineal or penile trauma, or iatrogenic needle injury. This condition leads to the formation of an arteriolacunar fistula, causing unregulated arterial blood flow into the sinusoidal spaces of the penis. Unlike low-flow priapism, high-flow priapism typically presents with a partially erect, non-painful penis. The diagnosis is confirmed through characteristic findings on color Doppler ultrasound, which reveals turbulent high-velocity flow pinpointing the fistula’s location. Blood gas analysis typically reflects arterial values, helping to differentiate high-flow priapism from its low-flow counterpart. Although high-flow priapism was historically considered non-urgent, recent evidence suggests that delayed treatment may increase the risk of erectile dysfunction. Therefore, prompt intervention by urologists is crucial. The primary goal is to close the fistula, and the treatment plan should be individualized based on the severity and duration of the condition. Urologists play a critical role in managing this condition, offering a range of therapeutic options. These include conservative approaches, such as observation and compression, medical therapy, arterial embolization, and, in some cases, surgical intervention. The choice of treatment depends on the patient’s condition, the fistula’s location, and the resources available. By ensuring timely and appropriate management, urologists can minimize complications and preserve erectile function.

## Introduction

Priapism is defined as a persistent or prolonged erection without sexual stimulation and does not subside [1]. There are three different types of priapism: low-flow (veno-occlusive, ischemic), high-flow (arterial, non-ishemic) and stuttering (recurrent or intermittent) [1].

High-flow priapism (HFP) (arterial) is a prolonged erection caused by irregular cavernous arterial flow. Typically, the penis is not fully erect and not painful [2]. It can occur following blunt perineal trauma, iatrogenic needle injury or shunt procedures for management of low-flow priapism [2]. Ultimately, arterio-sinusoidal fistula develops. Fistulas are mostly due to perineal trauma and occur proximally [2]. Hypoxia, hypercarbia, or acidosis is not observed in the blood gases of the corpus cavernosum [3, 4].

HFP, though rare, presents a unique challenge in urology, demanding specialized expertise for effective management. The role of the urologist in addressing this condition is multifaceted, encompassing both diagnostic acumen and therapeutic proficiency. Central to their responsibility is the accurate identification of HFP, often through meticulous history-taking and diagnostic imaging techniques. Once diagnosed, the urologist orchestrates a tailored treatment plan, which may involve conservative measures such as observation or, in more severe cases, invasive interventions like selective arterial embolization [1]. In cases where conservative and minimally invasive approaches prove ineffective, the urologist may also resort to open surgical techniques for managing HFP [1].

The aim of this review is to summarize the role of the urologist in the management of HFP, a condition with limited data in the literature, and to examine the treatment options and recent developments.

## Method

Articles focusing on the diagnosis and treatment of HFP were searched in MEDLINE and PubMed published until December 2023. The search terms used were “high flow priapism”, “non ischemic priapism”, “selective arterial embolization”, “prolonged erection” and “priapism”. All papers identified were English‐language papers.

## Epidemiology

The incidence of priapism is quite low across all types, with rates reported as 0.5–0.9 per 100,000 per year. [5]. In another study, the incidence of low-flow priapism is up to 5.34 per 100,000 men per year [6]. HFP is detected much less frequently than low-flow priapism, approximately 5% of all priapism cases [1, 6].

## Etiology and pathophysiology

The cavernosal artery enters the hilum of the penis and continues distally within the corpus cavernosum. Along its course, it gives off numerous twisted terminal branches known as helicine arteries [7]. These arterioles terminate in small and narrowed sinusoidal spaces. In the flaccid state, the smooth muscles of the arterioles and sinusoids are contracted. This state allows only a small amount of blood to enter the penis for nourishment in the flaccid state [8]. As the sinusoidal spaces expand and arterial blood flow begins to increase, resistance decreases [8]. In the flaccid state, the diameter of the cavernosal artery and terminal arteries are 0.05 cm and 30 µm,respectively. Whereas in the filling phase, the diameter of the cavernosal artery and terminal arteries increases to 0.1 cm and 100 µm, respectively [8, 9].

HFP typically occurs as a result of blunt perineal/penile trauma or iatrogenic needle injury, leading to unregulated arterial blood flow into the sinusoidal space due to the formation of a fistula between the artery and lacunar spaces [10]. HFP is characterized by irregular arterial blood flow to the corpus cavernosum, typically from the cavernous artery itself or a damaged branch of it [11]. Prolonged erections typically manifest within 2–3 weeks following the trauma or as a result of nocturnal erections or sexual activity [10, 12].

Nitric oxide (NO), which is produced both in cavernosal nerves and endothelium, has been recognized to play a key role in the physiology of penile erection [13]. The formation of NO occurs through the regulatory effects of the enzyme nitric oxide synthase (NOS) in the nitrergic nerves and endothelial cells covering the corporal smooth muscle cells and helicine arteries. The structural forms of the enzyme, neuronal NOS (nNOS), and endothelial nitric oxide synthase (eNOS), are the key NOS isoforms involved in inducing penile erection [13]. In HFP, there is no corporal relaxation associated with nNOS, resulting in the absence of a full erection. Increased arterial blood flow due to fistula leads to increased high oxygen levels and increased release of eNOS, consequently causing an incomplete, semi-rigid erection [13, 14]. Nonetheless, with some erectile stimuli or nocturnal erections, increased corporal smooth muscle relaxation can occur, leading to a priapism [15, 16]. In an in vitro study reported by Wessells and colleagues, it was demonstrated that increased fluid shear stress is associated with increased release of NO from endothelial cells and enhanced phosphorylation of eNOS [17]. Metaxa et al. also showed that high shear stress (more than 50 dyn/cm2) stimulates NO production by increasing eNOS in endothelial cells [18].

In a study reported by Kuefer et al. trauma-related or iatrogenic HFP accounted for 70.5% of the cases [19]. While HFP is often associated with trauma, it can also be induced iatrogenically during the treatment of low-flow priapism [20]. Additionally, there is a theory that similar to cardiometabolic pathologies the ischemic phase may trigger an excessive release of vasodilatory factors and reactive hyperemia [21]. The risk of HFP following shunt procedures for low-flow priapism appears to be correlated with the number of injections, irrigations, and shunt procedures performed on the patient [22–24]. HFP can also manifest in patients with spinal cord injuries, likely due to an increase in parasympathomimetic effects and enhanced arterial flow resulting from the loss of sympathetic impulses [25]. The exact mechanism behind HFP in patients with sickle cell anemia has not been definitively established. Instances of HFP with increased arterial flow, occurring without a history of trauma or fistula, have been reported in patients with sickle cell anemia [12, 26]. On an occasional basis, HFP has been documented following procedures such as internal urethrotomy, circumcision, the Nesbit procedure, transrectal prostate biopsy, and brachytherapy for prostate cancer [27–30].

Traditionally, HFP was not regarded as a urologic emergency, and spontaneous resolution was observed in most of cases [31, 32]. Erectile dysfunction (ED) has been reported in cases with HFP [12, 31]. Nevertheless, recent studies have demonstrated that, in contrast to the conventional perspective, excessive oxygen levels can result in corporal smooth muscle necrosis and subsequent ED in cases of HFP based on studies showing that high oxygen levels cause necrosis in other tissues [33]. The normal pO2 value is between 20 and 40 mmHg when the penis is flaccid, and this value rises to 100 mmHg during erection [34]. In HFP, the corpus cavernosum is exposed to high pO2 levels due to persistent arteriovenous fistulae. Cell culture experiments demonstrate that cells, particularly when exposed to 40% or higher oxygen levels, tend to damage. Exposure to hyperoxia is associated with increased free oxygen radicals, which consequently lead to cellular damage [33]. In a study by Zacharakis and colleagues, it is concluded that supraphysiological pO2 levels within the corpus cavernosum, along with continuous perfusion, lead to the formation of reactive oxygen species (ROS). These free radicals are thought to cause apoptosis and fibrosis in the smooth muscle of the corpus cavernosum [35]. Furthermore, in a study using an in vitro priapism model, it was demonstrated that supraphysiological pO2 levels (95% O2) resulted in a progressive decrease in rabbit corpus cavernosum smooth muscle tone [3]. Therefore, prompt treatment of HFP is essential (Table 1).

**Table 1:** Main points of the management of high flow priapism.

High flow is priapism is not an emergency, but it should be treated as soon as possible.
Perineal compression with elastic bandage may assist in promoting the spontaneous closure of the existing fistula.
Medical treatments (androgen deprivation therapy) are focused on supporting fistula closure, primarily by preventing nocturnal erections.
Selective arterial embolization can be performed if conservative therapy is failed. Repeat embolization procedure can be performed.
Open surgery can be performed in selected cases after multiple arterial embolization procedures.

## Diagnostic evaluation

Patients with HFP seldom present with complete erection and pain. Typically, they experience incomplete rigidity that causes little or no discomfort [1]. It’s important to obtain a history of perineal, pelvic, or genital trauma occurring a few weeks prior to the examination [1]. During the physical examination, the penis may not be fully rigid, and signs of trauma should be assessed during pelvic and genital examinations (butterfly sign, etc) [36]. A neurological examination should also be performed to identify underlying neurological pathologies such as spinal cord injury, transverse myelitis. Laboratory tests should include a complete blood count, white blood cell count, and coagulation parameters to rule out hematologic pathologies. Corporal aspirate appears bright and oxygenated, and blood gas analysis in corporal aspirate should be conducted to rule out low flow priapism. Blood gas measurements in cases of HFP are consistent with arterial blood gas values (pO2 > 90, pCO2 < 40, pH = 7.4) (refs. 5, 16, 31). At the onset of prolonged erection, arterial flow is increased and it should be noted that the blood gas obtained in the first few hours of priapism may not exhibit the typical hypoxia and acidosis findings [2, 37]. Different characteristics of low and high flow priapism are demonstared in Table 2.

**Table 2:** Differences between low and high flow priapism.

	Ischemic/low-flow	Non-ischemic/high flow
*Etiology*	SCD, hematological diseases, neoplastic syndromes and several pharmacological agents	Perineal or penile trauma, iatrogenic needle injury
*Symptom*	Painful, rigid erection	Painless, semi-erection
*Penile blood gas analysis*	Dark blood, low pO2 levels, High pCO2 levels, asidosis	Bright red arteriosus blood, and has high pO2 level
*Penil Doppler USG*	Decreased or absence of blood flow in the cavernosal arteries	Localize the fistula site and appears as a characteristic color blush and turbulent high-velocity flow

*SCD* Sickle cell disease, *pO2* Partial pressure of oxygen, *pCO2* Partial pressure of carbon dioxide, *USG* ultrasonography.

**Fig. 1:**
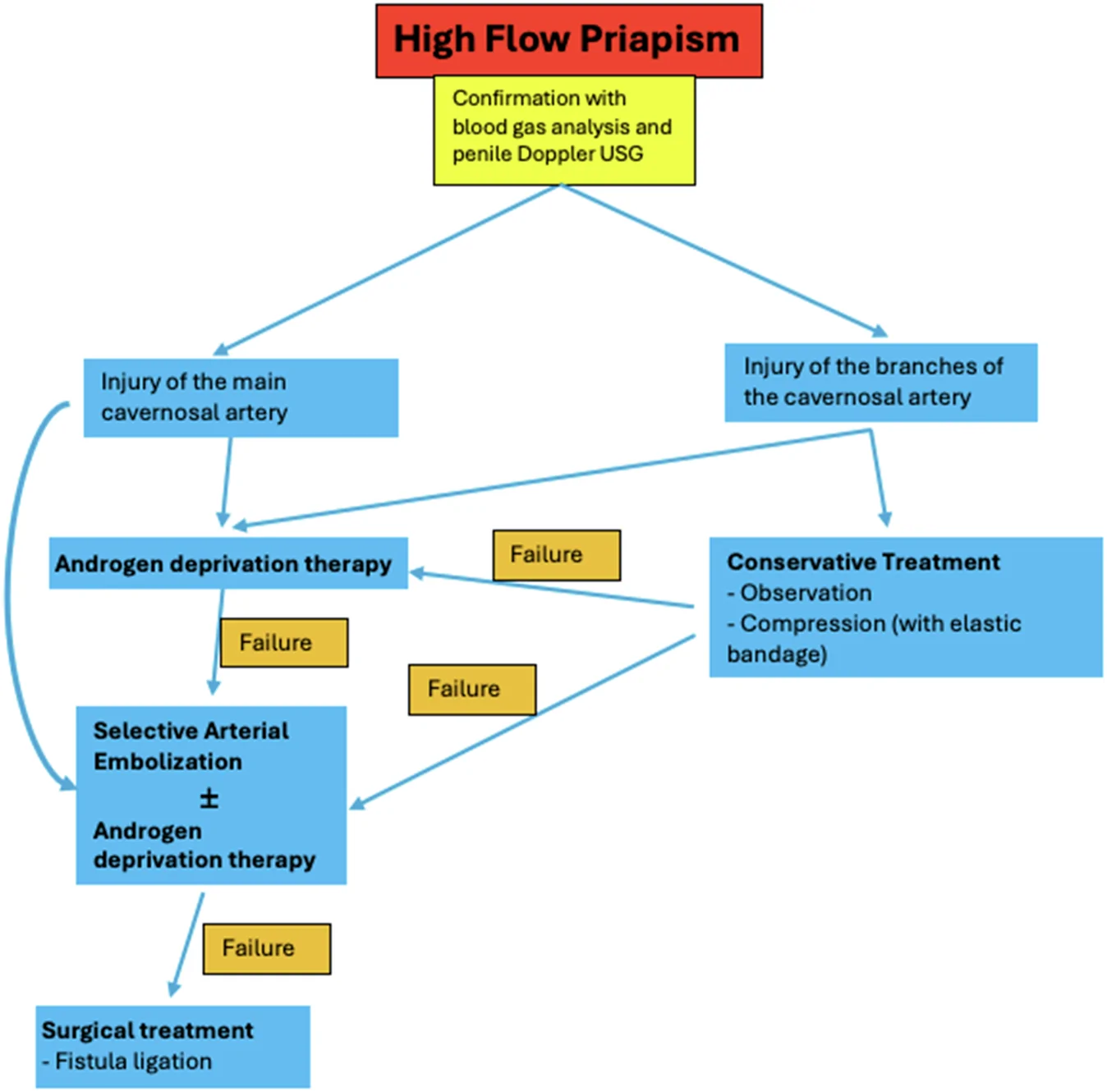
Management algorithm of high flow priapism.

Color Doppler ultrasound (CDUS) is a valuable tool for evaluating the penis and perineum. The characteristic turbulent high-velocity flow, indicating the location of the fistula, can be detected with ultrasound guidance. The sensitivity of CDUS in localizing an arterio- cavernosal fistula is nearly 100% (ref. 38). The injury is identified in the cavernosal artery or its branches using CDUS, contributing to the treatment strategy [10] (Fig. 1). CDUS is generally effective in distinguishing between low-flow and high-flow priapism. However, its usefulness is limited in the very early stages of priapism, where the differences in blood flow characteristics between the two types may not be pronounced enough to provide a clear diagnosis [39]. In a study, the hemodynamic outcomes of both low-flow and high-flow priapism were demonstrated in patients experiencing priapism for up to seven hours after intracavernosal injection. Specifically, in low-flow iatrogenic priapism lasting over 20 h, there was a loss of cavernosal arterial flow [40]. During these early stages, other diagnostic tools, such as blood gas analysis, might be required to accurately differentiate the types of priapism.

In cases of HFP resulting from pelvic trauma, contrast-enhanced CT is recommended for evaluating associated conditions, although it is not effective for diagnosing priapism itself [32].

The role of MRI in the diagnostic evaluation of priapism remains controversial. Its utility in HFP is restricted due to the challenge of visualizing the small penile vessels and fistulae [41]. MRI is advantageous in demonstrating nonviable smooth muscles after low-flow priapism and in detecting unusual conditions such as malignant infiltration [42].

The diagnosis of a fistula can be confirmed through pudendal arteriography. It’s important to note that arteriography is an invasive procedure and should only be performed in patients who are planned for embolization in the context of HFP [32].

In summary, the sensitivity of CDUS in localizing an arterio- cavernosal fistula is nearly 100% and CDUS is also preferred in follow-up of these patients. CDUS is non-invasive and widely available but can be operator-dependent. Angiography remains the gold standard for diagnosing and managing HFP due to its direct visualization of arterial blood flow and capability for therapeutic intervention. However, it is an invasive procedure with associated risks.

## Disease management

While it has been traditionally believed that HFP may not require immediate treatment, prolonged HFP can lead to ED [43]. Increased arterial blood flow and excessively high oxygen levels can result in apoptosis of corporal smooth muscle cells following HFP, affecting up to 30–40% of patients. Consequently, the treatment of HFP should not be delayed (Table 1). While the development of ED following HFP is not expected, patients with distal corporal fibrosis and ED requiring pharmacotherapy or penile prostheses have been reported [35, 44]. Close monitoring of patients using CDUS and penile MRI can aid in the detection of distal fibrosis and may prove useful in making clinical decisions for earlier intervention. The primary objective of treatment is to close the fistula, and to achieve this goal, various treatment options such as conservative/medical approaches, arterial embolization, or surgical intervention can be considered.

### Conservative-medical treatment

Unlike low-flow priapism, both the American Urological Association (AUA) and the European Association of Urology (EAU) guidelines recommend conservative medical therapies as the primary treatment for HFP [1, 31]. Interventions like aspiration, irrigation, and sympathomimetic drug injections are not recommended for the treatment of HFP. Perineal ice application and compression may assist in promoting the spontaneous closure of the existing fistula (Table 1). These techniques aim to induce vasospasm in the cavernous artery, potentially leading to thrombosis and the obstruction of the fistula. In a study, conservative management of HFP led to the resolution in two of seven patients (28%) (ref. 45). Moreover, wrapping the penis with a bandage can prevent recurrent priapism by preventing nocturnal erections, which may otherwise contribute to clot resolution and reformation of fistulae (Table 3).

**Table 3:** Different treatment options for high flow priapism.

Treatment Option	Efficacy	Success rate	Complications	ED	References
*Conservative/Medical*	Variable; often not curative	28–62%	None reported or minimal	NA	[1, 19, 20, 31, 45, 47]
*Arterial Embolization*	High, often effective in closing fistula	80–100%	Risk of arterial injury, thrombosis, infection, or embolization of non-target vessels	15–20%	[5, 32, 47, 53, 54]
*Surgical Intervention*	High, especially in cases of failed embolization or persistent priapism	63%	Risk of infection, bleeding, or recurrence	0–50%	[16, 31, 32, 54]

*ED* erectile dysfunction, *NA* non avaliable.

There is insufficient research available on how follow-up should be conducted in conservative treatment. In a review, it has been suggested that patients can be observed for six weeks, with physical examination and CDUS checks every two weeks [46].

It has been reported that spontaneous detumescence develops in most of the patients [19, 20, 31]. In cases of HFP, medical treatments are focused on supporting fistula closure, primarily by preventing nocturnal erections [47] (Fig. 1). This can be achieved by suppressing the hypothalamo-pituitary-gonadal axis and reducing androgen levels, thereby decreasing or preventing nocturnal erections. Androgen deprivation therapy options include daily ketoconazole (200 mg) + prednisone (5 mg) or long-acting 7.5 mg leuprolide acetate injections or 50 mg daily oral bicalutamide therapy [2, 47, 48]. It’s important to note that androgen deprivation therapy may lead to side effects such as decreased sexual desire, fatigue, hot flashes, gynecomastia, and ED [49]. After the intake of 200 mg of ketoconazole, one of the agents used in androgen deprivation therapy, total and free testosterone levels drop below 60% of the baseline within 6–8 h and return to baseline values within 24 h [50, 51]. Therefore, for the treatment of HFP, ketoconazole is recommended once daily for a duration of six months [50, 51]. Small arterial bleeding occurring in the branches of the cavernous artery can lead to thrombosis through vasoconstriction or external pressure. Androgen deprivation therapy eliminates nocturnal erections, minimizing blood flow in the bleeding branch and facilitating clot formation in the ruptured area. However, if this situation occurs within the main cavernous artery, the success of compression and androgen blockade treatment is diminished [2].

While intracavernous methylene blue injection has been attempted to prevent HFP by inhibiting NO-mediated smooth muscle relaxation, it has not yielded successful results [12, 52].

### Selective arterial embolization

**Fig. 2:**
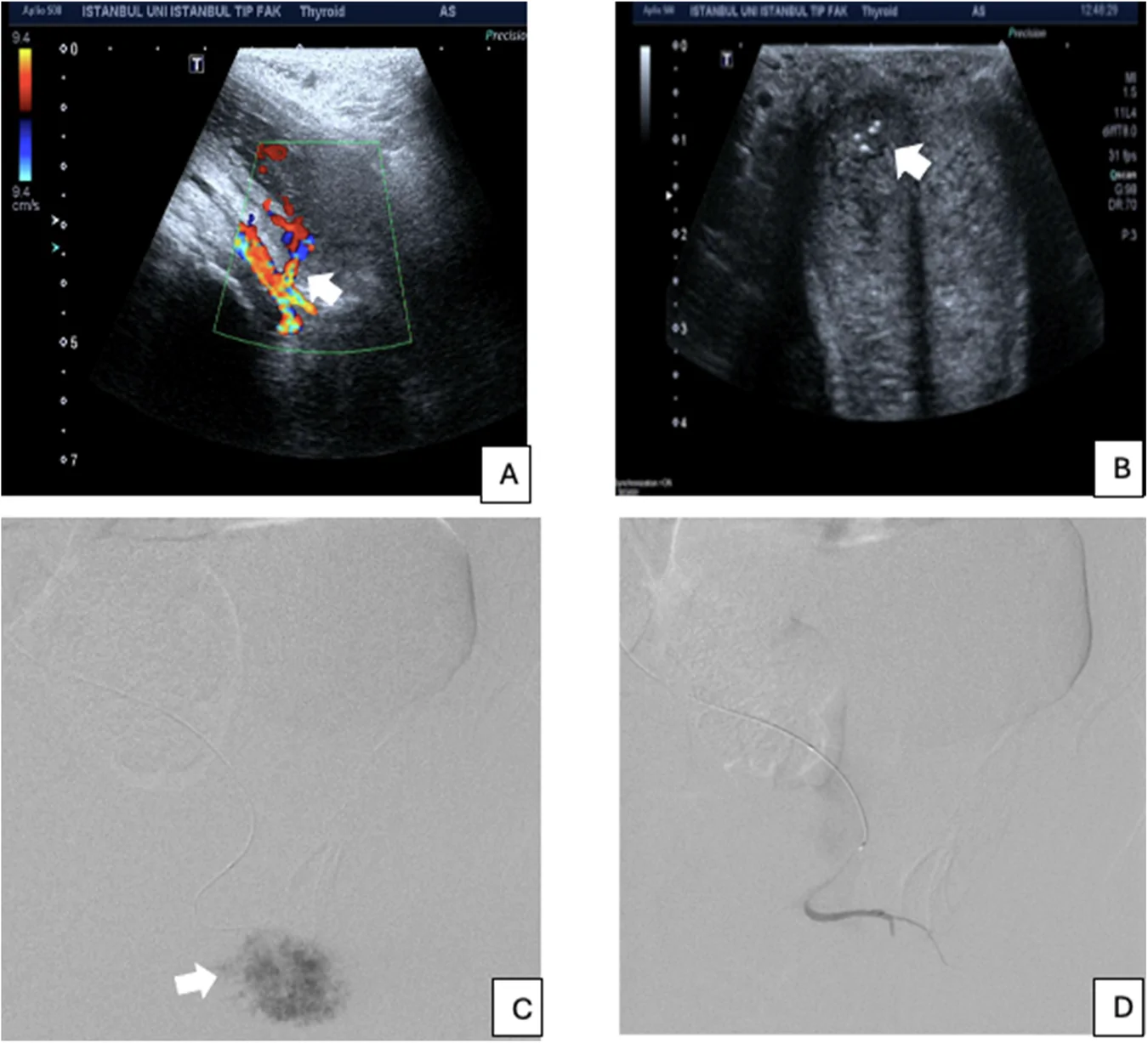
Pre- and post-treatment imaging in a patient with high-flow priapism. **A** Color Doppler image showing a fistula with turbulent flow (arrow); **B** Coronal B mode ultrasound image demonstrating no evidence of fistula. Hyperechoic foci (arrow) are seen, representing dispersed gelatin sponge particles within lacunar spaces, a finding associated with embolization in early ultrasound follow-u; **C** Selective angiogram of the right cavernosal arteries demonstrating an arteriocavernous fistula (arrow); **D** Selective angiogram after absorbable gelatin sponge embolization demonstrating successful embolization of the arteriocavernous fistula (Archive of İstanbul Faculty of Medicine).

In selective arterial embolization treatment, the aim is to close the fistula with temporary (such as autologous blood clot and gel foam) or permanent (such as microcoils, ethylene-vinyl alcohol co-polymer and N-butyl-cyanoacrylate) materials [32, 53]. Selective arterial embolization has approximately 80–100% success and a 6–21% recurrence rate has been reported [5, 32, 54] (Table 3). This treatment may have side effects such as penile gangrene, gluteal ischemia, cavernositis and perineal abscess if embolizastion is performed proximally than appropriate area. ED may occur in 15–20% of patients after this treatment. The preference of embolization type and embolic agent has not been shown to be superior to each other in terms of treatment success and side effects [32]. Figure 2 shows CDUS and angiografic imagines of a patient who underwent selective arterial embolization for HFP.

Hovewer, some of the studies reported that ED risk is greater when permanent embolization materials such as coils are used. Also, the use of autologous blood clot does not completely change the risk of ED [47].

In cases of HFP in which the initial embolization procedure failed, successful results have been reported with repeated procedures [55, 56]. In a study reported by Volkmer and colleagues, initial successful embolization was achieved in 22 out of 27 patients (81.4%). Among the five patients for whom the initial procedure was unsuccessful, a second embolization resulted in success for three patients (60%) (ref. 57). Compressing the penis with a bandage after the embolization procedure may enhance its success and reduce recurrence. Androgen deprivation therapy may also contribute to success by eliminating nocturnal erections, thereby minimizing blood flow to the bleeding branch and increasing the efficacy of embolization in the ruptured area [2] (Fig. 1).

### Surgical management

According to the guidelines provided by the AUA and the EAU, open surgery is only contemplated as a last resort after multiple arterial embolization attempts [1, 31]. This surgical method involves fistula ligation, boasting a success rate of up to 63% (ref. 31). Nonetheless, there is a 50% likelihood of ED development [16] (Table 3). Waiting for pseudocapsule formation to aid in fistula identification can be beneficial, but one should consider that pseudocapsule formation may take weeks or even months. The aim of the surgery involves the ligation of the internal pudental artery, cavernous artery or its branch supplying the corpus cavernosum, resulting in impaired erectile function. The ligation could be performed through ingunoscrotal approach. After inguinoscrotal incision, the shaft of the penis is dissected from the superficial portion of the Buck’s fascia. Partially lowering the suspensory ligaments reveals the proximal dorsal neurovascular bundle of the penis. With optical magnification, after visualizing the bifurcation of the penile and cavernosal arteries entering the crus distally, the cavernosal artery is ligated. Subsequently, a decrease in corporal blood flow velocity is observed with CDUS, resulting in detumescence. [58]. In an old study, ligation of one internal artery was reported resulting in prompt reduction of the priapism without loss of erectile function [59]. Recently surgical treatment has become less prevalent, likely due to advancements in interventional radiology and the availability of various embolization agents [32, 54] (Table 1).

Open surgery is technically challenging, and when there are contraindications for embolization or if repeated embolization fails, surgery should be considered. If the patient seeks a more radical solution or has pre-existing ED or is not sexually active, surgery may be an appropriate option. Despite all treatments, long-term penile prosthesis implantation may be performed in patients [60].

Urologists play a critical role in the management of HFP, a rare form of priapism characterized by unregulated blood flow into the penile arteries. Their primary focus is on accurately diagnosing HFP, distinguishing it from the more common low-flow priapism through clinical evaluation and imaging techniques like CDUS. Once diagnosed, urologists work on managing the condition by promoting the closure of the underlying arteriovenous fistula, which is typically responsible for the unregulated blood flow. Urologists may prescribe medications to prevent nocturnal erections, as these can impede the healing process and prolong the condition. By carefully monitoring the patient’s progress and adjusting treatment plans as necessary, urologists ensure the best possible outcomes while minimizing the risk of complications like ED. Post-priapism, they manage ED through a combination of medical therapies, such as phosphodiesterase type 5 (PDE5) inhibitors, intracavernosal injections, or penile prosthesis implantation if necessary.

## Conclusion

HFP is not an emergency like low-flow priapism, but it should be treated as soon as possible. The treatment is stratified whether the ruptured side is in the cavernosal artery or in its branches. While conservative approaches are typically the initial step in treatment, the minimally invasive nature of selective arterial embolization makes it a suitable option to consider after conservative measures. In cases of unsuccessful embolization attempts, repeated procedures can be considered. Only in selected cases, surgical management can be performed (Fig. 1).
